# Supplementary figures and images for: Normalization in mouse primary visual cortex
Source: PLoS One. 2023 Dec 18;18(12):e0295140. doi: 10.1371/journal.pone.0295140 (PMC10727357; doi:10.1371/journal.pone.0295140)

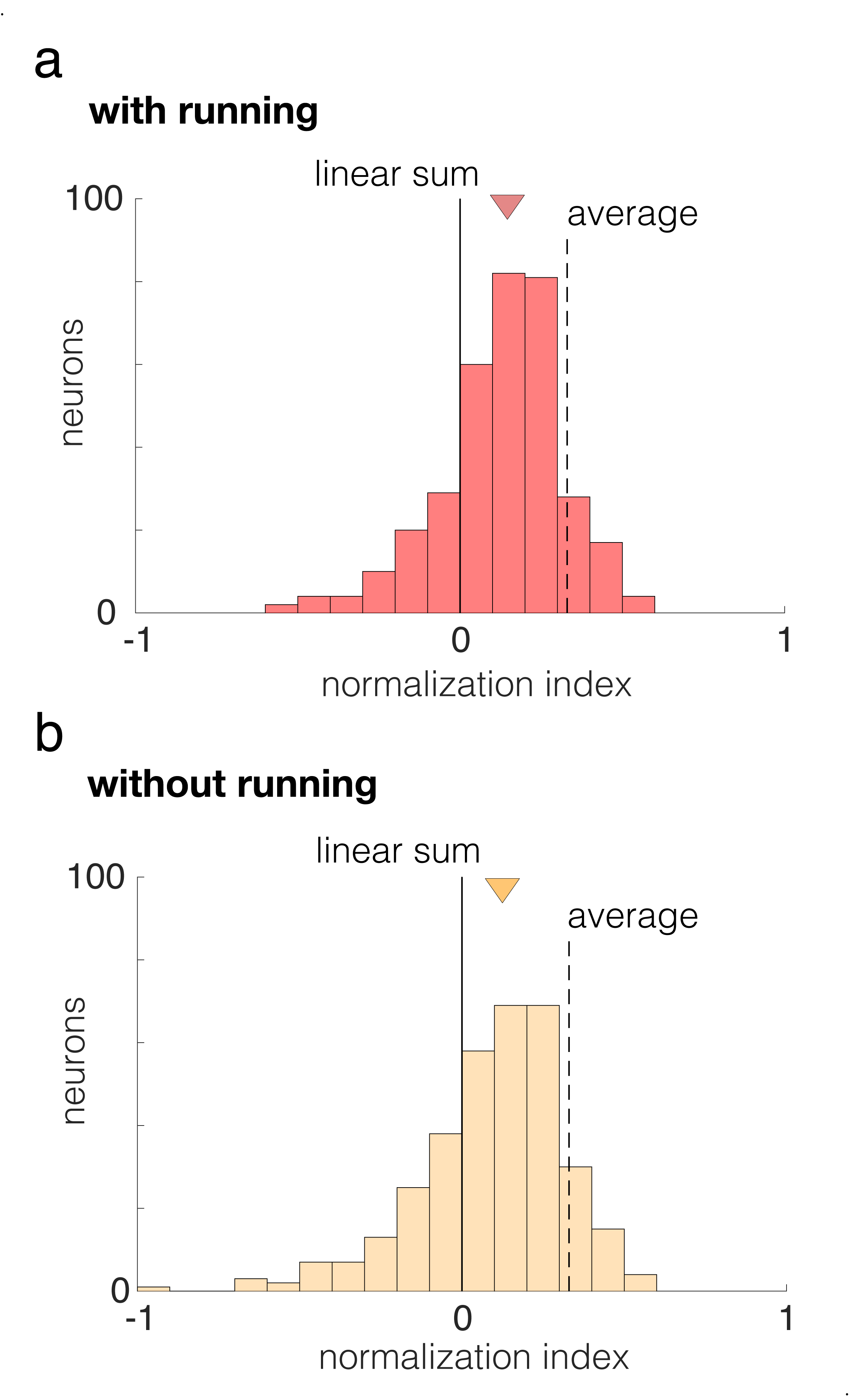

Supplement: S1 Fig — a, Normalization index of neurons recorded during optophysiology experiments (n = 341). The triangle represents the median (median = 0.15, IQR = 0.03–0.25; bootstrapped median 95% CI = 0.13–0.17). b, Normalization index measured in the same neurons as in S1A Fig, after excluding trials in which mice ran. The triangle represents the median (0.13, IQR = -0.02–0.25, bootstrapped median 95% CI = 0.10–0.15). No difference in the medians or overall distribution of normalization indices was statistically detectable (Mann-Whitney U test, n.s.; two-sample Kolmogorov-Smirnov test, n.s.). A normalization index of 0.00 represents the expected normalization index for linear summation and the dashed line at 0.33 represents the expected normalization index for simple averaging. (TIF) [file pone.0295140.s001.tif]
